# The potential of substance P to initiate and perpetuate cortical spreading depression (CSD) in rat in vivo

**DOI:** 10.1038/s41598-018-36330-2

**Published:** 2018-12-05

**Authors:** Frank Richter, Annett Eitner, Johannes Leuchtweis, Reinhard Bauer, Andrea Ebersberger, Alfred Lehmenkühler, Hans-Georg Schaible

**Affiliations:** 1 Institute of Physiology I/Neurophysiology, Jena University Hospital - Friedrich Schiller University Jena, Jena, Germany; 2 Institute of Molecular Cell Biology, Jena University Hospital - Friedrich Schiller University Jena, Jena, Germany; 3 Pain Institute and Center for Medical Education, Düsseldorf, Germany

## Abstract

The tachykinin substance P (SP) increases neuronal excitability, participates in homeostatic control, but induces brain oedema after stroke or trauma. We asked whether SP is able to induce cortical spreading depression (CSD) which often aggravates stroke-induced pathology. In anesthetized rats we applied SP (10^−5^, 10^−6^, 10^−7^, or 10^−8^ mol/L) to a restricted cortical area and recorded CSDs there and in remote non-treated areas using microelectrodes. SP was either applied in artificial cerebrospinal fluid (ACSF), or in aqua to perform a preconditioning. Plasma extravasation in cortical grey matter was assessed with Evans Blue. Only SP dissolved in aqua induced self-regenerating CSDs. SP dissolved in ACSF did not ignite CSDs even when excitability was increased by acetate-preconditioning. Aqua alone elicited as few CSDs as the lowest concentration of SP. Local pretreatment with 250 nmol/L of a neurokinin 1 receptor antagonist prevented the SP-induced plasma extravasation, the initiation of CSDs by 10^−5^ mol/L SP diluted in aqua, and the initiation of CSDs by aqua alone, but did not suppress KCl-induced CSD. Thus neurokinin 1 receptor antagonists may be used to explore the involvement of SP in CSDs in clinical studies.

## Introduction

The principal excitatory transmitter in the brain is glutamate^1^, and the principal inhibitory transmitter is gamma-amino butyric acid (GABA)^2^. However, many neurons in mammal central nervous system (CNS) also contain substance P (SP), a neuropeptide often released as a cotransmitter from glutamatergic and GABAergic neurons. SP is contained in whole cerebral cortex, hippocampus, basal ganglia, hypothalamus, in trigeminal sensory neurons^3^, in primary sensory neurons, and in the dorsal horn of the spinal cord^4^. Correspondingly, the receptor of SP, the NK-1 receptor (NK-1R), is expressed in the CNS on neurons, glial cells, and endothelial cells^5^. SP can also bind to the NK-2 and NK-3 receptors, but with less affinity than the neurokinins A or B, respectively^4^. In rat and guinea pig cerebral cortex the NK-1R is preferentially expressed in superficial layers^6,7^, and many of these SP-positive neurons were identified as interneurons containing the transmitter GABA^8,9^.

SP increases neuronal membrane excitability^10^. It is involved in physiological functions in the normal/healthy brain but also in pathophysiological functions. SP contributes to the regulation of stress responses^11,12^ and nociception^13^, participates in the regulation of depression and anxiety^11,13^, and causes nausea and vomiting, e.g. during motion sickness^14^. The most deleterious function of SP in CNS is the induction of plasma extravasation and the mediation of vasogenic oedema^15–17^ after traumatic brain injury^17,18^ or after stroke^16^. SP participates in the development of an inflammatory response in the brain after such injury including activation of microglia and release of proinflammatory cytokines^19,20^. The increase in neuronal excitability caused by SP raises the issue whether SP has a proconvulsant action^21^.

Given the large variety of putative functions of SP ranging from an involvement in physiological to severe pathophysiological functions, the question arises which effects are elicited by SP alone, and which effects of SP may be dependent on the pathophysiological context. Since SP evokes neuronal as well as vascular effects, it may be important, therefore, to determine whether neuronal or vascular components or both are involved in a pathophysiological event. Either reduced blood supply due to a disturbed diffusion process or vice versa enhanced oxygen consumption due to pathological increased neuronal activity can induce the same net result, a reduced brain tissue oxygenation.

Since stroke and traumatic brain injury are associated with the release of SP, the pathophysiological mechanism of cortical spreading depression (CSD) comes into focus. Propagating CSD waves which often occur after stroke or traumatic brain injury^22,23^ also cause the release of SP^24^. A CSD is a mass depolarization of neurons and glial cells^25–27^ accompanied  by a transient change in ion and water distribution between extra- and intracellular space^28,29^. The redistribution of ions and water after a CSD is energy demanding. This is not a serious challenge in normoxic tissue^28^, but can cause cytotoxic oedema and neuronal death in metabolically compromised brain tissue via long-lasting and/or repeated ionic redistributions and CSD-related hypoperfusion (spreading ischemia)^29,30^. Though it is known that SP can depolarize neurons^10^ and induce bursts of action potentials in pyramidal cells of the entorhinal cortex^9^ it has not been investigated yet whether such depolarizing effects may also induce CSD. Therefore we explored in the present study whether SP lowers the threshold for CSD elicited by a depolarizing stimulus (KCl). We tested whether SP triggers CSD by itself, or needs an additional conditioning stimulus. Well-known conditioning stimuli are the replacement of a part of the extracellular chloride ion content by acetate or osmotic challenges. An isoosmolar replacement of 75% of chloride by superfusing the brain with artificial cerebrospinal fluid (ACSF) containing an equimolar amount of acetate (acetate-ACSF) enhances neuronal excitability and renders brain areas susceptible to SD that are usually resistant against it, such as cerebellum^31^, spinal cord^32^, immature rat cortex^33^ or immature rat brain stem^34^. Osmotic challenges such as hypoosmolarity are also known to change the susceptibility of brain tissue for CSD^35,36^. We applied SP topically to a small area of the exposed cortex at different concentrations and we administered SP following pretreatment with a NK-1R antagonist. SP was applied either in physiological artificial cerebrospinal fluid (ACSF), or in ACSF following acetate-preconditioning, or it was applied diluted in aqua.

## Results

**Figure 1:**
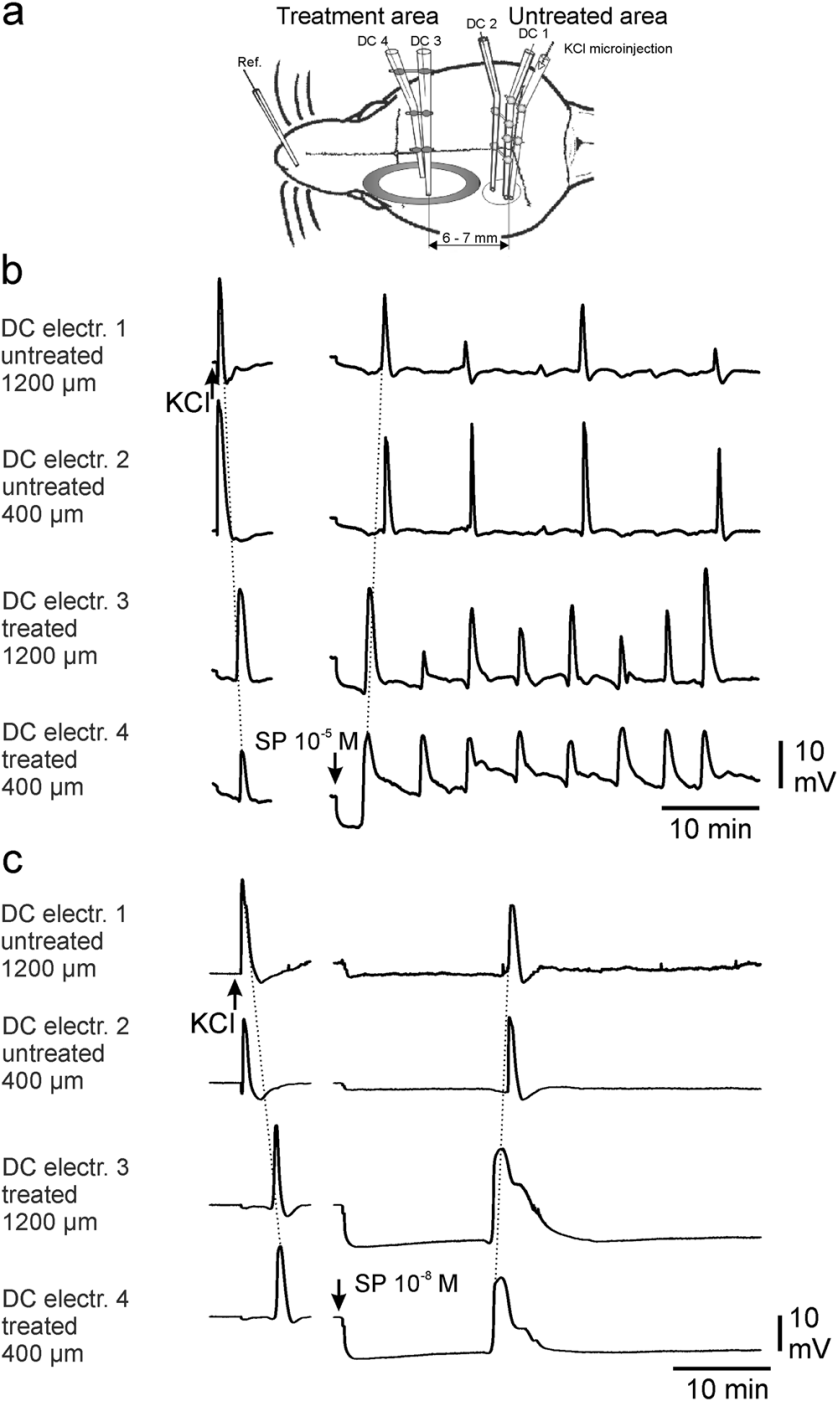
Experimental design and typical samples of SP-induced CSDs. Schematic top view (**a**) on the skull of the rat with the position of the recording electrodes. CSDs after either application of SP at 10^−5^ mol/L (**b**) or 10^−8^ mol/L (**c**) dissolved in aqua. The separated traces in the left sides of each panel show the control CSDs elicited by KCl microinjection. Note the propagation from electrode 1 to 4 when KCl is injected versus propagation from electrode 4 to 1 when SP is applied. The propagation direction of the CSDs is accentuated by oblique thin dotted lines.

In order to check whether the cortex is capable to generate CSDs, two CSDs were elicited at the beginning of each experiment at intervals of 20–30 min by KCl microinjection at electrodes DC 1 and DC 2 while the exposed cortex was superfused with ACSF. In all rats one microinjection evoked a single propagating CSD wave with amplitudes up to 23 mV that moved from the site of microinjection at a velocity of 2.3 to 2.8 mm/min and was  picked up at all 4 recording electrodes (Fig. 1).

### CSDs after topical application of substance P (SP) onto the cortical surface

#### a) Elicitation of CSDs by topical application of SP

In 3 rats we applied 100 µL of 10^−5^ mol/L SP dissolved in ACSF into the trough of the treatment area and did not see CSD-related DC deflections within an observation time of 2 hrs. The application of 100 µL of 10^−6^ mol/L SP dissolved in ACSF was tested in 1 rat and did not induce any DC-deflections either. Thus even at high concentrations SP did not elicit CSDs when applied in physiological ACSF.

Preconditioning the brain by exchanging chloride ions by acetate has been used successfully in the past to render brain areas susceptible for SD that are in the native state resistant against SD, such as the cerebellum^31^ or the brainstem^34,37^. Therefore in another 3 rats, we first enhanced neuronal excitability in the cortical treatment area by the local superfusion of acetate-ACSF for two hours. The subsequent topical administration of 100 µL of 10^−6^ mol/L SP dissolved in ACSF did not induce CSDs either, thus showing that SP is not a sufficient stimulus for CSD even after enhancing excitability by acetate-preconditioning.

Since hypoosmolar solutions induce hyperexcitability in hippocampal slices^38,39^ we used aqua instead of ACSF to dissolve SP, and we applied SP in aqua to the treatment area. We tested 4 different concentrations of SP dissolved in aqua (10^−5^; 10^−6^; 10^−7^; 10^−8^ mol/L, respectively) and monitored the number of CSD waves for 2 hours. Figure 1 shows two examples from experiments with either 10^−5^ mol/L or 10^−8^ mol/L SP dissolved in aqua. The initial KCl-application elicited typical single CSD. Following application of SP in aqua either a series of CSDs or more sustained negative deflections were observed. In most cases we observed the typical CSD-shape with durations of the negative deflections from 40 to 120 s (“classical CSD”). In 10 out of 24 experiments we found rather sustained negative DC deflections (see below and Table 1). The CSDs propagated from the treated brain area into the remote cortical area with a propagation velocity of 2.3 to 2.9 mm/min.

**Table 1:** CSD amplitudes (top) and CSD duration at half-maximal amplitude (bottom) in the treated area during controls elicited by KCl before (Ctrl 1) or at the end of application of SP (Ctrl 2), and during application of SP dissolved in aqua or application of aqua only (SP n = 6 rats each, aqua n = 4 rats), numbers in brackets in the top table show numbers of CSD waves at this cortical depth over all rats tested. Bold numbers in the lower table indicate statistically significant differences to Ctrl 1 (paired Student’s t-test).

	400 µm cortical depth: CSD amplitude [mV]				1200 µm cortical depth: CSD amplitude [mV]			
	Ctrl 1	First two CSD by test subst.	All CSD during test subst.	Ctrl 2	Ctrl 1	First two CSD by test subst.	All CSD during test subst.	Ctrl 2
SP 10^−5^ mol/L	17.5 ± 2.6	20.9 ± 1.3	18.1 ± 1.2 (32)	21.1 ± 2.8	21.9 ± 1.3	22.0 ± 1.4	20.5 ± 1.3 (32)	24.8 ± 1.1
SP 10^−6^ mol/L	20.9 ± 1.2	26.2 ± 1.4	27.0 ± 0.8 (44)	22.0 ± 4.2	22.5 ± 1.3	24.1 ± 1.8	26.2 ± 1.0 (41)	20.9 ± 4.8
SP 10^−7^ mol/L	20.1 ± 1.1	21.9 ± 2.5	22.7 ± 1.8 (20)	25.8 ± 1.8	21.0 ± 1.3	20.1 ± 2.6	22.7 ± 1.0 (18)	25.5 ± 0.8
SP 10^−8^ mol/L	16.7 ± 3.0	19.0 ± 1.8	19.0 ± 1.8 (9)	17.7 ± 4.7	18.1 ± 3.4	22.7 ± 2.6	20.2 ± 2.9 (12)	17.0 ± 4.5
Aqua	20.9 ± 0.5	22.0 ± 1.9	22.5 ± 1.3 (10)	26.1 ± 2.1	22.9 ± 0.7	22.2 ± 2.4	23.5 ± 1.8 (10)	25.0 ± 1.7
	**400 µm cortical depth: CSD duration at ½ amplitude [s]**				**1200 µm cortical depth: CSD duration at ½ amplitude [s]**			
	**Ctrl 1**	**First two CSD by test subst**.	**All CSD during test subst**.	**Ctrl 2**	**Ctrl 1**	**First two CSD by test subst**.	**All CSD during test subst**.	**Ctrl 2**
SP 10^−5^ mol/L	28.6 ± 2.8	**72.9** ± **16.2** (p = 0.0135)	**58.0** ± **7.4** (p = 0.0208)	**40.2** ± **5.0** (p = 0.0405)	31.4 ± 2.4	74.9 ± 19.6	77.7 ± 15.5	**45.3** ± **5.4** (p = 0.0132)
SP 10^−6^ mol/L	32.7 ± 2.6	**86.8** ± **22.5** (p = 0.0262)	50.3 ± 7.3	**56.2** ± **8.2** (p = 0.0029)	29.2 ± 1.8	**65.6** ± **9.3** (p = 0.0332)	72.6 ± 17.8	**43.5** ± **5.4** (p = 0.0063)
SP 10^−7^ mol/L	35.6 ± 3.2	39.4 ± 5.6	45.3 ± 6.3	**114.5** ± **45.7** (p = 0.024)	27.4 ± 3.0	34.0 ± 2.4	31.5 ± 1.6	60.7 ± 22.9
SP 10^−8^ mol/L	56.2 ± 12.4	73.8 ± 12.5	73.8 ± 9.3	55.2 ± 13.4	30.0 ± 1.7	**117.6** ± **27.6** (p = 0.0004)	**117.6** ± **24.3** (p = 0.0004)	**44.7** ± **6.8** (p = 0.0134)
Aqua	27.3 ± 1.8	**83.7** ± **13.1** (p < 0.0001)	**83.7** ± **13.1** (p < 0.0001)	28.5 ± 2.9	30.3 ± 5.9	**103.9** ± **37.5** (p < 0.0001)	**103.9** ± **37.5** (p < 0.0001)	25.4 ± 1.8

**Figure 2:**
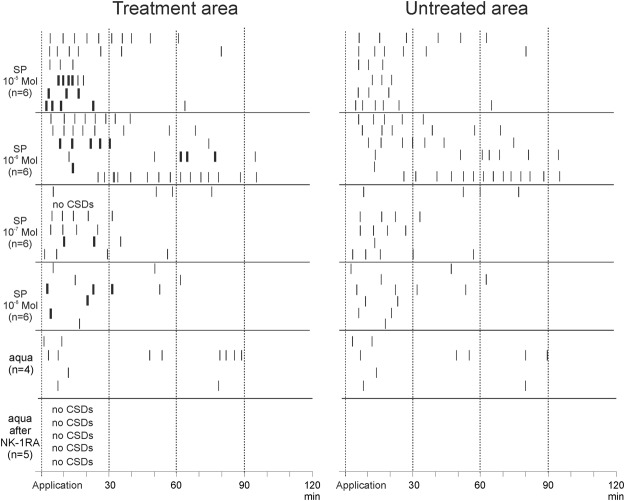
Occurrence of CSD events after topical application of either SP dissolved in aqua at different concentrations or aqua alone to the treatment area. The left panel shows the CSDs in the treated area, the right panel shows the respective CSDs in the untreated area. Each row represents the data from one animal, each bar represents the peak time of one single CSD wave. Thin bars show classical CSDs, thick bars sustained CSD events. Note that no sustained CSDs occurred in the untreated area.

Figure 2 displays the patterns of CSDs observed in the treated area (left panel) and in the untreated area (right panel) after SP application. Each line shows the CSDs in one experiment. Classical CSDs are displayed by thin bars, sustained negative deflections by thick bars. The first CSD started at least 1.5 min and at the latest 24 min after SP application. In all animals except one CSDs were observed after SP treatment. The number of CSDs was dose-dependent with preponderance in the first 30 min of application. In the first 30 min of application a dose of 10^−5^ mol/L SP and over the whole observation time a dose of 10^−6^ mol/L SP elicited significantly more recurring CSD waves than doses of 10^−8^ mol/L (p = 0.0043, p = 0.0213, respectively; one-way ANOVA). In one animal 10^−7^ mol/L SP did not elicit any CSD (empty line in Fig. 2), but the microinjection of KCl still evoked a single CSD before and after SP application. The amplitudes of the CSDs during SP application did not differ markedly from those elicited with KCl during controls (Table 1). As mentioned in Materials and Methods, the putative internalization of NK-1R allowed us to assess only the effect of one dose of SP in a particular experiment, and it excluded the testing of a higher dose if the initial dose had no effect.

In Fig. 1 it can be seen that the application of SP in aqua caused an immediate positive shift of the ECoG baseline between 6 and 12 mV both at 400 µm and at 1200 µm of cortical depths. The magnitude of the shift did not significantly differ between the four concentrations of SP. The shifts reached maximal amplitudes after 100 to 120 s, and could usually be seen at decreasing amplitudes throughout the application of aqua to the treatment area.

In order to exclude that the solvent aqua was responsible for the elicitation of CSDs we applied in 4 rats 100 µL aqua without SP topically to the treatment area. Application of aqua alone induced the same positive shift of the ECoG baseline as aqua + SP (between 5 and 10 mV at both cortical depths, reaching deepest values after 40 to 390 s). In the first 30 min after aqua application one propagating CSD wave was observed in a time interval of 119 s (minimum) to 707 s (maximum) in all 4 rats, and in two rats a second CSD was observed, but only in one rat this second CSD propagated into the untreated area (Fig. 2). After aqua CSDs propagated significantly faster from the treated area into the remote cortical area that was superfused with ACSF (3.7 ± 0.4 mm/min, during first controls 2.6 ± 0.2 mm/min; p = 0.0342; Wilcoxon signed-rank test). Altogether the number of CSDs ignited by aqua was significantly less (p = 0.0043; one-way ANOVA) than the number of CSDs after 10^−5^ mol/L SP in aqua. The number of CSDs ignited by aqua did not differ from the number of CSDs after 10^−8^ mol/L SP dissolved in aqua. We conclude therefore, that only the application of SP at a minimum dose of 10^−7^ mol/L caused series of recurring CSD waves. However, since SP in ACSF did not elicit CSDs, the conditioning by aqua was necessary to elicit repetitive CSDs.

#### b) Characterization of the CSDs observed during application of SP and KCl

In the treated area the CSD waves after SP application had insignificantly larger amplitudes than CSDs during controls. The first one or two CSD waves lasted significantly longer at half of maximal amplitude at 10^−5^ and 10^−6^ mol/L SP (Table 1). By contrast, the CSD elicited by KCl at the end of the recording time had similar amplitude and duration as the CSD elicited by the initial KCl injection.

We also tested, whether SP in aqua changed elicitability of CSDs and the thresholds (injection time at constant pressure) to elicit CSDs by KCl microinjection. SP was applied in 24 rats and KCl-thresholds were unchanged in 22 rats. In two rats (one rat tested with 10^−5^ mol/L SP, one rat tested with 10^−8^ mol/L SP) the threshold increased to a threefold KCl injection time for eliciting a propagating CSD. Application of aqua alone in all rats tested in this group (n = 4) had no influence on CSD elicitability by KCl, each microinjection elicited one single CSD, and the thresholds for KCl microinjection did not change.

**Figure 3:**
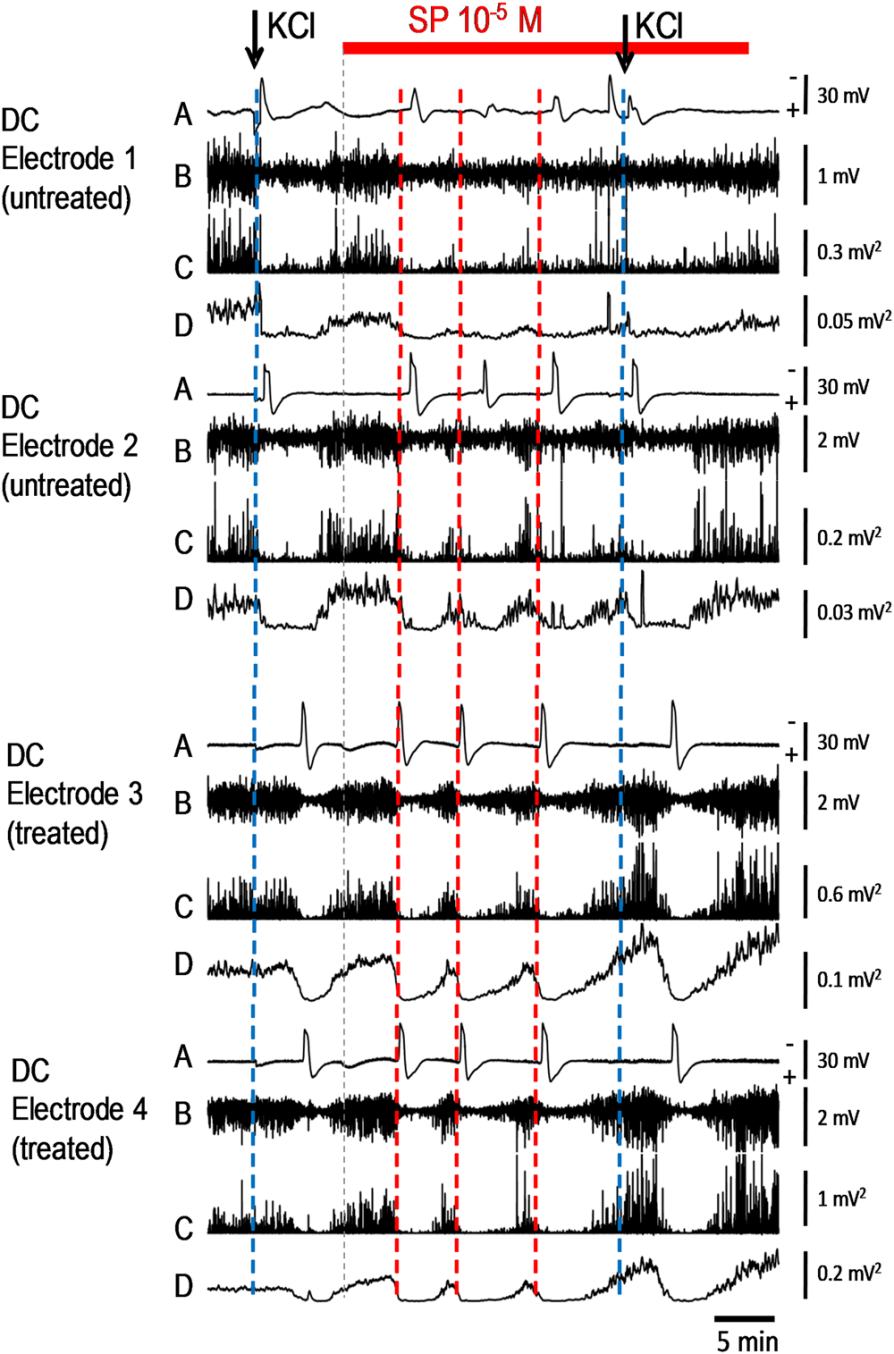
Original intracortical DC-ECoG recordings and filtering of the DC-ECoG recordings to obtain the AC-ECoG during KCl- and SP-evoked CSDs. Single KCl-induced CSDs (application marked by arrows) started at DC electrode 1 (accentuated by blue dotted lines) and moved to electrodes 3/4. Self-regenerating CSDs ignited by SP at 10^−5^ mol/L started at DC electrode 3 or 4 (accentuated by red dotted lines) and moved to electrodes 1 and 2. Analysis revealed that in both cases real “spreading depressions of EEG activity” occurred: **A** Recordings of spreading depression-related negative shifts in the DC recordings, negative shifts are facing upwards; **B** high-pass filtered ECoG data with a lower frequency limit of 0.5 Hz of the respective recordings, **C** power of bandpass filtered recordings (0.5–45 Hz), and **D** integral of power of bandpass filtered recordings C (0.5–45 Hz) respectively, which allow the visualization of the spreading depressions in the ECoG recordings. Note that the application of the hypoosmolar solution (indicated by the beginning of the red bar, the thin dotted line and the small positive shifts in traces A of electrodes 3 and 4) had no effect on the AC-ECoG pattern.

In order to compare the CSDs elicited by KCl and SP we also analysed the ECoG recordings (Fig. 3). A similarity between SP- and KCl-induced CSDs was revealed by the high-pass filtering (lower frequency limit 0.5 Hz) of the recorded raw direct current (DC) ECoG. The application of the hypoosmolar SP-solution did not change the AC-ECoG pattern unless a CSD started. During all CSDs the alternate current (AC)-ECoG was depressed and the depression spread across the cerebral cortex. In KCl-induced CSD the depression started at DC electrode 1 where KCl was injected into the grey matter, and moved in frontal direction to electrodes 3/4. By contrast, the self-regenerating CSDs during application of SP dissolved in aqua at 10^−5^ mol/L started at DC electrode 3 or 4 and moved to electrodes 1 and 2. The reconstruction from the DC recordings was confirmed by the decrease in power of the AC-ECoG, i.e. the local brain activity over time, or in the integral in power of the AC-ECoG that diminished down to zero during a CSD wave at the particular recording electrode. No differences were seen regarding the first CSD wave (probably ignited by low osmolarity) and the subsequent ones.

**Figure 4:**
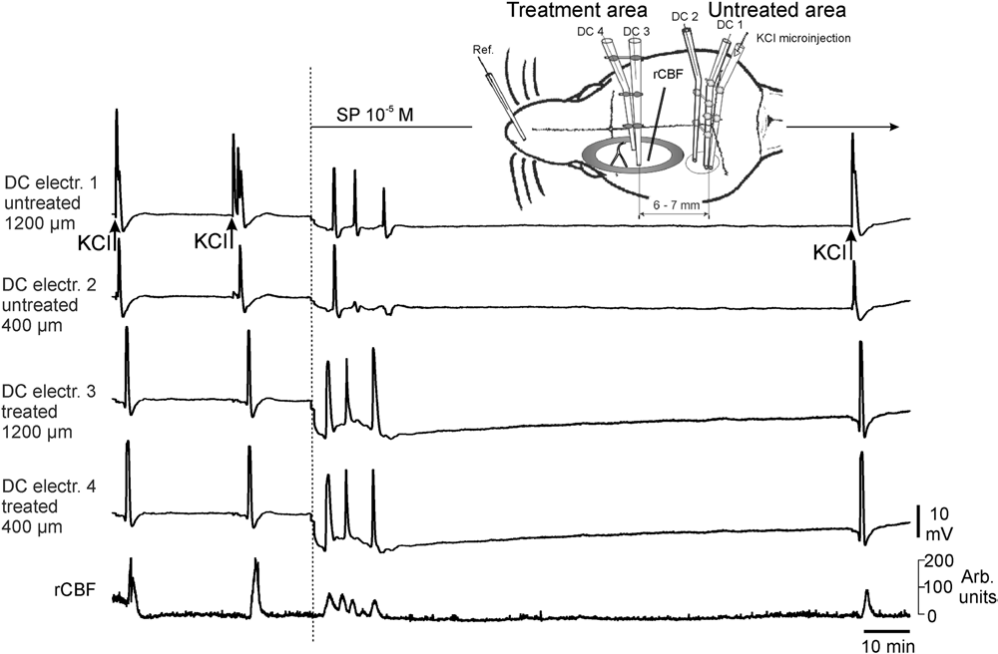
Traces of a complete experiment with simultaneous recording of the regional cerebral blood flow (rCBF) in the SP treated brain area. Far left the control period is shown in which the cortex was superfused with ACSF and CSDs were elicited by KCl. Then SP at 10^−5^ mol/L dissolved in aqua was applied and evoked a series of three CSD waves at the DC electrodes 4, 3, and 1, only one typical wave at electrode 2, but four events in the rCBF recording thus further underlining that not all CSD events were picked up at all recording sites.

Each typical CSD wave was accompanied by an increase in regional cerebral blood flow (rCBF). The rCBF increased during CSD by KCl to 168.0 ± 18.5% and significantly smaller to only 136.1 ± 3.9% (p = 0.0254; Student’s t-test) during CSDs by 10^−5^ mol/L SP (Fig. 4). The duration of the increases in rCBF did not differ significantly (CSDs by KCl 126.6 ± 12.7 s; CSDs by SP 105.1 ± 12.3 s). The laser probe was not exactly at the same place as the recording electrodes in the treated area. Therefore the starting points of the change in rCBF and the beginning of the CSD wave did not exactly correspond. In none of the experiments changes in rCBF occurred without a CSD-related DC shift and vice versa.

**Figure 5:**
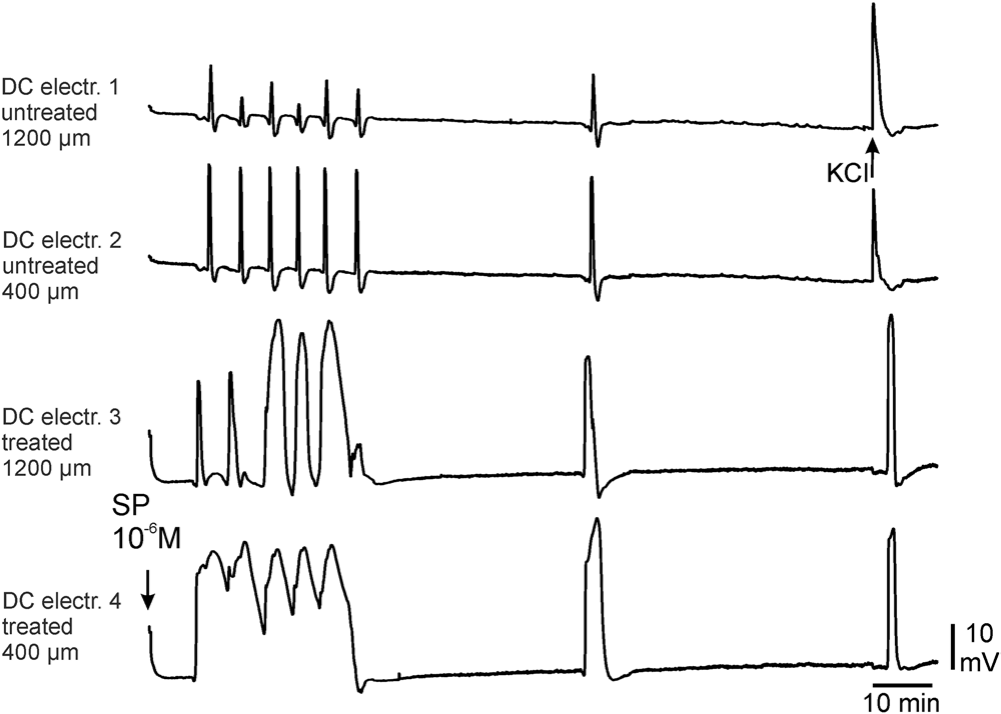
Sustained negative shift observed at DC electrode 4 after 10^−6^ mol/L of SP dissolved in aqua. The positive shift of the EEG baseline was initiated when SP was applied. Note the sequence of five negative DC deflections at electrode 4 starting about 8 min after application of SP that did not completely return to baseline whereas in the untreated brain area six CSD waves can be clearly distinguished. The last CSD far right is elicited by KCl.

Interestingly, as already mentioned, some sustained negative DC shifts instead of classical CSD waves were seen in each concentration of SP in aqua (see example in Fig. 5). We observed 10 sustained DC shifts (3 at 10^−5^ mol/L SP, 3 at 10^−6^ mol/L SP, one at 10^−7^ mol/L SP, 3 at 10^−8^ mol/L SP) that lasted longer (min. 289 s; max. 1666 s at half-maximal amplitude) than the longest CSD-related DC deflection by aqua (194.5 s), and occurred only in the treatment area. When only aqua was applied to the cortical surface, the CSD waves were broadened (see also Table 1), but in no case a sustained negative shift occurred. Sustained DC shifts had the same rising times as classical CSDs until the first peak and reached similar amplitudes (mean values at 400 µm 25.6 ± 2.3 mV; at 1200 µm 26.2 ± 1.9 mV). They were observed in both cortical depths of the treatment area, and lasted significantly longer before the DC potential returned to baseline values. From sustained DC shifts single CSD waves propagated into the untreated area (Fig. 5, see also Fig. 2), thus showing that this negative deflection of the DC potential was not a terminal depolarization (Fig. 5). All sustained shifts slowly returned to baseline, and in five animals (one at 10^−5^ mol/L SP, 3 at 10^−6^ mol/L SP, one at 10^−7^ mol/L SP), after recovery from the sustained negative shift, single CSD waves propagated from the treated area into the remote one while SP dissolved in aqua was still onto the cortical surface. Thus sustained negative DC shifts did not indicate a persistent deleterious condition.

### Blocking the NK-1 receptor prevents the occurrence of CSD waves induced by SP dissolved in aqua and prevents the ignition of CSD waves by aqua alone

**Figure 6:**
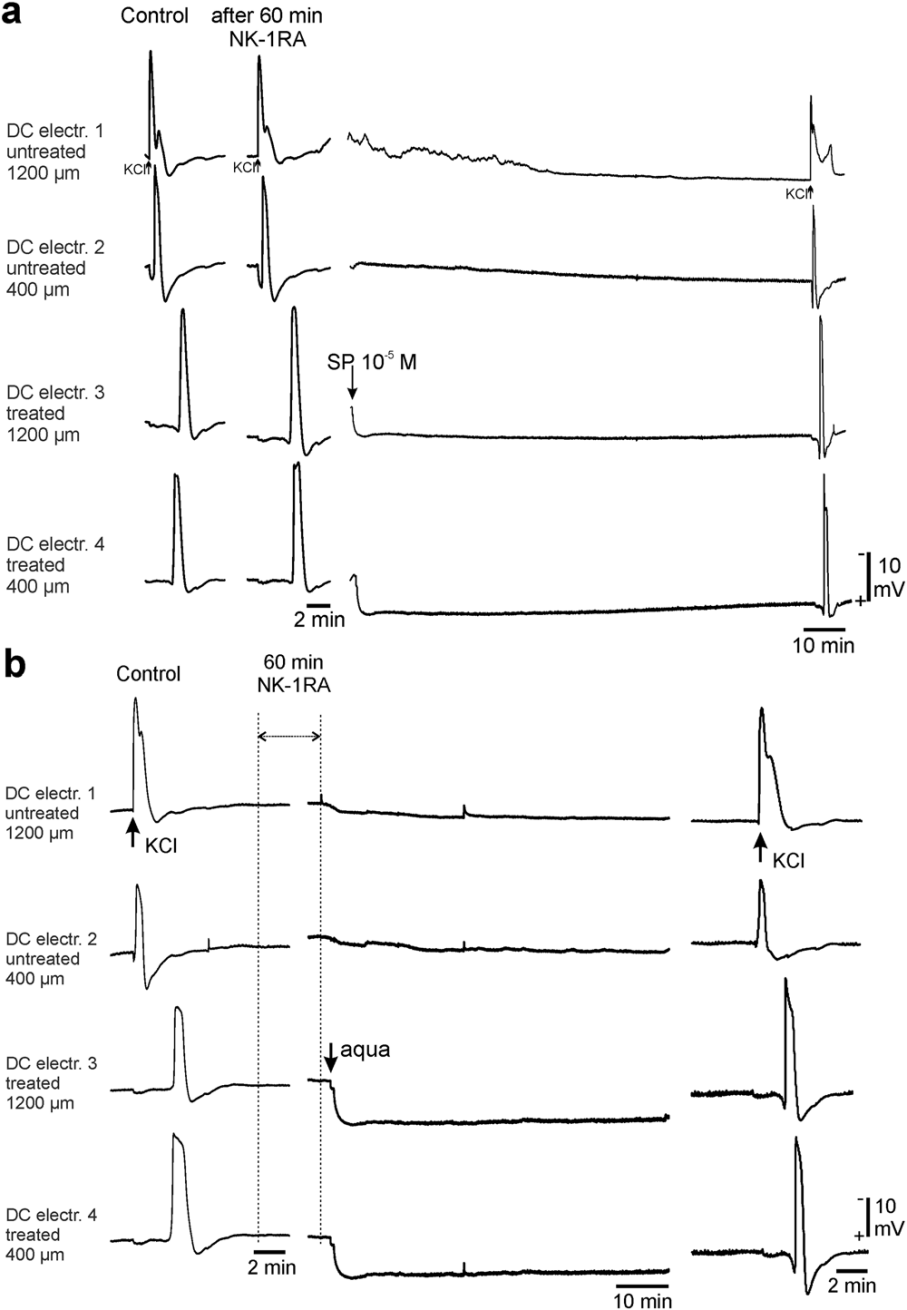
Pretreatment with the NK-1RA L-703,606 prevented the occurrence of CSDs ignited by SP or by aqua alone. (**a**) The traces show from left to right the control CSD by KCl in the native cortex, as well as a CSD by KCl after one hour pretreatment with the NK-1RA and the two hour observation phase when 10^−5^ mol/L of SP dissolved in aqua was applied to the treated area. The CSD far right is elicited by KCl. (**b**) The traces show from left to right the control CSD by KCl in the native cortex, the beginning and the end of one hour of blocking the NK-1R and the complete phase when aqua was applied. No CSDs occurred in the time range when the NK-1R was blocked. The CSD far right is elicited by KCl.

In order to test whether the CSDs evoked by SP involved an action specifically at the NK-1R, we applied in a group of five rats for one hour an antagonist to the NK-1R (L-706,303, Sigma-Aldrich) (NK-1RA) at 250 nmol/L topically to the cortical surface. The NK-1RA did not interfere with CSD waves elicited by KCl. Each KCl-microinjection during NK1R-blockade elicited one CSD wave. The CSD-amplitudes in the treated area were the same as during controls, and the propagation velocity did not differ (Fig. 6A).

After removal of the NK-1RA we applied SP dissolved in aqua at 10^−5^ mol/L to the treatment area. In none of these 5 rats SP in aqua elicited any CSD wave. The subsequent microinjection of KCl evoked a typical CSD which did not differ from those during controls. Interestingly, the propagation velocity increased significantly (p = 0.0395; Wilcoxon  signed-rank test) from 2.7 ± 0.1 mm/min during controls to 4.0 ± 0.5 mm/min at the end of the experiment when we tested for CSD after blocking the NK-1R and SP application. The threshold for CSD elicitation by KCl was unchanged (Fig. 6A).

In another five rats we found that the blockade of the NK-1R even prevented the ignition of CSDs by aqua alone. In three rats we first enhanced neuronal activity by superfusion with acetate-ACSF for two hours, and then we applied the NK-1RA for one hour. The application of aqua after blocking the NK-1R did not induce any CSDs. The same result we found in two other rats without an acetate-ACSF preconditioning (Fig. 6B).

### Plasma extravasation induced by substance P

Though the topical application of SP dissolved in ACSF did not induce DC shifts (see above), the medial cerebral arteries showed a slight swelling and the cortical surface was stained reddish in a small rim adjacent to the vessels after the application of SP for 2 hrs.

**Figure 7:**
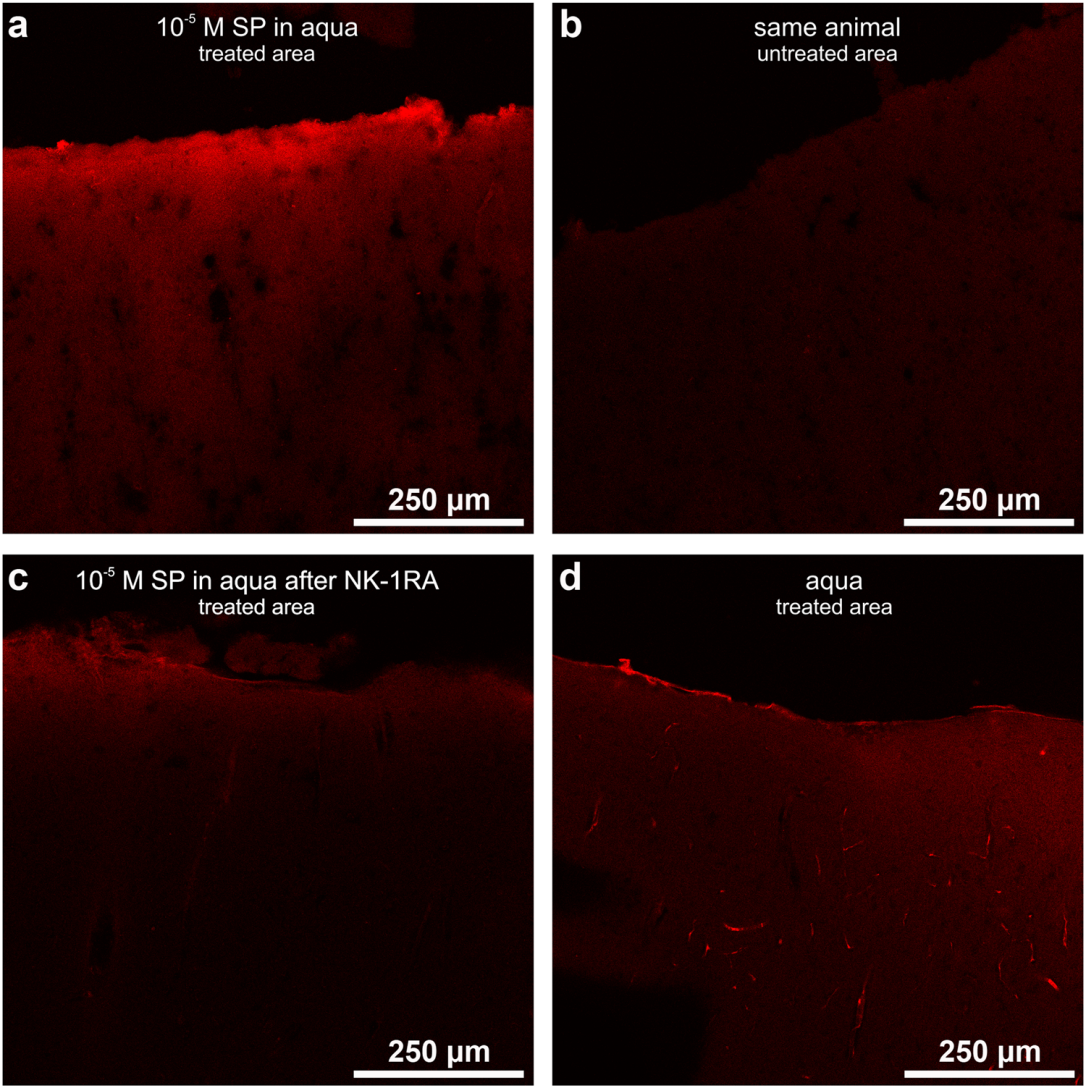
Laser scanning photomicrographs of cortical slices after injection with Evans Blue displaying plasma extravasation. (**a**) Plasma extravasation in the superficial layers of the cerebral cortex after 2 hours of topical application of 10^−5^ mol/L SP dissolved in aqua that induced self-regenerating CSD waves. (**b**) No plasma extravasation in the remote (untreated) cortical areas of the same animal. (**c**) No plasma extravasation occurred when the NK-1R were blocked in a 1 hour pretreatment before application of 10^−5^ mol/L SP dissolved in aqua. (**d**) The application of aqua bidest. alone did not induce any plasma extravasation.

Application of 10^−5^ mol/L SP dissolved in aqua onto the surface of the exposed cerebral cortex resulted in a plasma extravasation that was even visible to the naked eye after two hours of SP application. Staining with Evans Blue confirmed this. The extravasation was more pronounced in the superficial cortical layers down to layer II (Fig. 7A) but was not observed in the untreated contralateral cortical hemisphere (Fig. 7B). When the NK-1R was blocked topically prior to the treatment with 10^−5^ mol/L SP dissolved in aqua no Evans Blue staining was visible in the SP-treated cortical area (Fig. 7C) thus confirming that the process of plasma extravasation is linked to functional NK-1R. The application of 100 µL aqua bidest. to the cortical surface rather caused a slight contraction of the medial cerebral arteries. Staining of the brain with Evans Blue showed no plasma extravasation in the treatment area (Fig. 7D).

## Discussion

In the present study we show for the first time that the topical application of SP to a small area of the cortex can ignite repetitive self-regenerating CSD waves. However, this effect was only observed if SP was applied as a solution in aqua and not if SP was dissolved in ACSF (even after acetate preconditioning). The effect of SP in aqua was dose-dependent since higher doses of SP evoked more CSD waves overall and longer series of self-regenerating CSD waves than lower doses of SP. The elicitation of self-regenerating CSD waves by SP as well as the elicitation of few CSDs by aqua alone was prevented by a preceding specific blockade of NK-1R. In addition, superfusion of the cortical area with SP elicited plasma extravasation in the superficial cortical layers. Thus, after preconditioning with aqua, SP may trigger repetitive self-regenerating CSDs which aggravate the pathophysiological processes.

Our conclusion that SP can evoke self-regenerating CSD waves under suitable conditions is based on two main findings. First, while the topical application of aqua alone induced only one or two CSD waves in the first 30 min of application, SP dissolved in aqua at doses of 10^−5^ or 10^−6^ mol/L elicited a series of self-regenerating CSDs in the same time range (a dose of 10^−7^ mol/L seemed to be the threshold dose under our experimental conditions). Second, following the pretreatment with the NK-1RA (in a dose adopted from Tao *et al*.^40^), SP dissolved in aqua (10^−5^ or 10^−6^ mol/L) did not elicit CSDs (though in one experiment subthreshold deflections of the cortical DC potential were seen). However, the NK-1RA did not impair the generation of CSD waves triggered by a microinjection of KCl. Together these findings support a specific effect of SP in triggering CSDs.

By acting on NK-1R SP depolarizes neurons^10^. Older studies showed that pyramidal neurons in rat and cat are excited by microiontophoretically applied SP^41–43^. This might be a paracrine effect of SP^44^. However, SP also reduces inward currents at GABA_A_ receptors, thus reducing activity of inhibitory neurons. Under suitable conditions (see below) the net effect might result in the observed mass depolarization (CSD).

However, the ignition of repetitive self-regenerating CSD waves was only observed when SP was applied to the cortex in aqua but not in ACSF. We had to exclude, therefore, that aqua itself caused the CSDs, e.g. by damaging the tissue. In fact, brain tissue damage is a major trigger of CSD^29,45–47^. The application of aqua to the cortical surface neither caused plasma extravasation nor changes of the AC-ECoG pattern in the observed time window. In contrast to SP in aqua the application of aqua alone to the cerebral cortex induced only single or very few CSDs. Therefore we conclude that the use of aqua as a solvent for SP did neither cause frank damage to the superfused cortex areas (see also^48^) nor induced consistently CSDs by itself. Aqua rather created a condition which enabled SP to elicit self-regenerating CSDs. As mentioned above, we did not observe the triggering of CSDs if NK1 receptors were blocked.

In brain tissue which is relatively resistant to the elicitation of CSD, the increase of excitability by acetate conditioning was found to facilitate the generation of CSDs. We therefore tested whether acetate preconditioning facilitated the generation of CSDs by SP similar as aqua. We applied acetate-ACSF first and then SP in ACSF. This protocol did not elicit self-regenerating CSD waves. It is unlikely, therefore, that aqua just increased the excitability to such a point that SP elicited CSDs.

An essential parameter of body fluids is their osmolarity and their content of specific ions. The application of aqua to the tissue is likely to induce a disturbance of the osmolarity and/or the composition of the fluids. It was reported that bathing hippocampal slices in hypoosmolar ACSF (Na^+^ content lowered by 60 or even 90 mMol to an osmolarity of 189 or 134 mosmol/kg, respectively) ignited self-regenerating SDs^38,39^. Furthermore, it was reported that hypoosmolar ACSF (180 mOsm) applied to hippocampal slices increased the volume of astrocytes by up to 12% within 5 min of application^49^. Astroglial swelling was also observed in cerebral cortex of mice *in vivo* when 150 mL/kg distilled water was injected i.p.^50^. Recent data show that not only astrocytes with AQP-4 aquaporins are able to swell. In cortical slices neurons swell even faster than astrocytes and increased their volume by 22 to 25% when hypotonic ACSF (180 mOsm) was applied. The swelling was attributed either to a water-permeable cotransporter or to a water flux across neuronal membranes^48^. According to Lauderdale *et al*.^49^ the neuronal swelling triggers slow inward currents (SICs) within 1 min after application of a hypoosmolar solution, and the SICs are nonsynaptic and depend on NMDA receptors. Volume-regulated anion channels should be involved in the generation of SICs and astrocytic Ca^++^ activity could modulate SICs^49^. Just to add a finding of the present study: The pretreatment with the NK-1RA prevented any CSDs by aqua alone suggesting that aqua may even elicit the release of SP.

Thus the literature shows that the disturbance of the local homoeostasis can elicit a plethora of different effects which could have preconditioned the exposed cortex for the effect of SP observed in the present study. However, while the recording of CSDs *in vivo* has many advantages (intact tissue in the natural surroundings, intact blood and energy supply etc.) this approach can hardly identify the particular conditioning mechanism(s) induced by aqua. It remains open, therefore, whether aqua conditioned the cortex for CSD just by inducing hypoosmolarity or whether other specific changes such as ion fluxes contributed to conditioning.

Regarding the recordings of CSD a particular observation of the present experiments must be addressed. After the application of aqua alone or after the application of SP in aqua a positive shift of the DC-ECoG baseline was noted. The shift persisted at decreasing amplitudes as long as aqua was in the trough over the treatment area, and removal of the aqua caused a baseline shift in the opposite direction. Such a positive shift was not observed when the same amount of isotonic solutions was applied. We assume that this shift is a technical artefact resulting from a difference in the ion concentration at recording and reference electrodes. Despite this positive shift spontaneously occurring CSDs were observed after SP in aqua in all experiments except of one, thus showing that the positive shift had not significantly elevated the threshold for CSDs elicited by KCl.

In addition to the electrophysiological recordings we measured plasma extravasation since SP induces plasma extravasation and vasogenic oedema^16,17,51^. At endothelial cells the response to SP is completely mediated via the NK-1R^15,52,53^. The topical application of the NK-1RA before the application of SP in aqua prevented the plasma extravasation confirming that NK-1RA antagonized SP effects. Importantly, the vasoactive actions of SP alone were not sufficient to trigger CSDs because SP dissolved in ACSF did not elicit self-regenerating CSDs. We assume that a normal extracellular space has the capacity to buffer excitatory neurotransmitters or excess potassium ions released by the excited neurons when SP dissolved in ACSF is applied.

Since the release of SP in the cortex is important in the context of stroke (see Introduction) we believe that the findings of the present study could have some clinical relevance. It is known that the release of SP after stroke or brain trauma fosters brain oedema and swelling causing secondary brain damages^16,17,20,54,55^. CSDs in the aftermath of stroke were shown to aggravate secondary brain damage^56^ and prevention of CSDs improved the prognosis for survival and revocery^57,58^. To our knowledge it has not been explored, neither clinically nor experimentally, whether the triggering of CSDs by SP contributes to the aggravation of the consequences of cytotoxic oedema and spreading ischemia in the compromised brain tissue^56^. The present data show that SP has the potential to trigger repetitive self-regenerating CSD waves under suitable conditions. In this respect it is interesting that the CSDs ignited by SP in aqua had a longer duration than KCl-induced CSDs and that even sustained negative DC shifts were observed, thus indicating a metabolic change in this brain area.

It is open, however, whether, and if, how the conditioning by aqua translates into the clinical situation. Furthermore, it is difficult to relate the concentrations of SP measured in human serum after stroke to the concentrations of SP in our experiments, since serum concentrations do not necessarily reflect the actual concentration in the brain which might be higher. The dose of 10^−7^ mol/L SP was in the range that was used in rat brain slices to depolarize nNOS-expressing neurons^6^. However, we could show in our experiments that NK-1RA blocks specifically SP-evoked CSDs, and therefore our study suggests that the application of an NK-1RA could be an important experimental tool to address the contribution of SP to disease-elicited CSDs. Such studies may reveal whether the triggering of CSDs by SP may particularly occur in brain damage which is associated with cytotoxic oedema and spreading ischemia in the compromised brain tissue^29,56^ or whether it may even be important in conditions such as migraine in which the brain does not exhibit damage.

## Materials and Methods

The present study was performed according to the Protection of Animals Act of the Federal Republic of Germany (Tierschutzgesetz der Bundesrepublik Deutschland) and was approved by the Thuringian State Office for Food Safety and Consumer Protection (Thüringer Landesamt für Verbraucherschutz, TLV). The animals were treated in accordance with the declaration of Helsinki and the guiding principles for the care and use of animals. Any effort was made to comply with the 3-R-guidelines and to reduce the number of animals used. Data sampling, evaluation, and presentation complied with the ARRIVE guidelines.

### Surgical preparation of the rats

Adult male Wistar rats (n = 44; 350–450 g, aged 95 ± 4 days, housed in the Animal Facility of University Hospital Jena) were deeply anesthetized with sodium thiopental (Trapanal; Inresa, Freiburg, Germany; initially 100–125 mg/kg intraperitoneally [i.p.]). During dissection, depth of anaesthesia was regularly assessed by testing for the absence of the corneal blink reflex and of reflexes to noxious squeezing of the interdigital skin. During the experiments, if necessary, supplemental doses at most of 20 mg/kg Trapanal i.p. maintained the depth of anaesthesia. The trachea and the right femoral vein and artery were cannulated. The mean arterial blood pressure and the electrocardiogram were continuously monitored. Body temperature was kept at 37 °C using a feedback-controlled heating system.

Surgical preparation of the skull was performed as previously described^59,60^. Briefly, the head was fixed in a stereotactic apparatus, two trephinations were made over the left hemisphere (one frontal, spanning from 2 mm in front of bregma over a length of 5–6 mm, 3–4 mm wide, and one circular more caudal, with a diameter of 3–4 mm in front of lambda) using a minidrill and cooling with ACSF. The composition of ACSF was in millimols per litre: NaCl 138.4, KCl 3.0, CaCl_2_ 1.3, MgCl_2_ 0.5, NaH_2_PO_4_ 0.5, urea 2.2, and glucose 3.4, warmed to 37 °C and equilibrated with 5% CO_2_ in O_2_. The underlying dura and arachnoidea were removed, and the exposed cortex was kept moist with ACSF. A barrier was built with dental acrylic on the skull around the large frontal trephination thereby forming a trough with a capacity of 100 to 150 µL for topical application of test compounds to a restricted cortical area (Fig. 1A). In a group of rats the composition of ACSF was modified to enhance neuronal excitability^31^ by replacing 75% of the Cl^−^ by sodium-acetate (acetate-ACSF). This acetate-ACSF was warmed and equilibrated as well. For conditioning the treated cortical area was superfused with acetate-ACSF for two hours.

### Recording of intracortical direct current potentials, regional cerebral blood flow, and data processing

An Ag/AgCl reference electrode (containing 2 mol/L KCl) was placed on the nasal bone. Electrodes for direct current (DC) electrocorticogram (ECoG) recordings had tip diameters of approximately 5 µm, resistance <10 MOhm, and were filled with 150 mmol/L NaCl. The electrode for CSD elicitation contained 1 mol/L KCl. SD waves were elicited by injection of 0.5 µL 1 mol/L KCl solution with a pressure of 100 kPa using a microinjector (picoinjector PLI-100; Harvard Apparatus, Holliston, MA, USA). Injection times ranged from 0.5 to 3 s. If the first KCl-microinjection of 0.5 s did not ignite a CSD, the injection time was increased in steps of 0.5 s. The intervals between the microinjections when a repetition was necessary lasted 3 to 5 min. The shortest injection time that elicited a CSD wave repeatable for two times was defined as threshold. The signals were recorded using a 4-channel high-impedance amplifier (Meyer, Munich, Germany) and stored on a personal computer (sampling rate 2.048 Hz). CSD were accepted if they had a steep onset between 1 and 3 s, reached amplitudes >5 mV, and propagated from electrode 1 to 4 or vice versa (Fig. 1A).

CSD were evaluated regarding occurrence in the treated areas, number of CSD events, time interval between application of the compound and occurrence of first CSD, maximal amplitudes related to baseline before the steep onset of the depolarization, duration at half-maximal amplitude and propagation time from the site of elicitation (electrode DC 1) to the deepest electrode in the treated area (electrode DC 3).

In each animal two CSD were elicited at intervals of 20 min by KCl microinjection prior to application of the test compounds to confirm the ability to produce CSD at all. Then SP was applied into the trough (see below). Another CSD was elicited by KCl at the end of the two hours of application of SP. In previous experiments we had shown that CSDs could be reproducibly evoked by microinjections of KCl at the same threshold (at least 7 CSD-events per experiment) over a time period of 6 hours^61^.

In a series of experiments the brain was topically pretreated with a NK-1RA for one hour. We induced CSD 30 min and 60 min after application of the antagonist. Then we applied SP into the trough, and another CSD was induced at the end of the SP application.

In order to analyse depression patterns of the ECoG activity associated with spreading depression, DC recordings were resampled offline with a sample rate of 205 Hz and first detrended by appropriate adaptive filtering, followed by band pass filtering (bandpass 0.01–45 Hz). In order to reveal alternate current (AC)-ECoG activity, the signals were high-pass filtered with a lower frequency limit of 0.5 Hz. In addition, power of bandpass filtered recordings (0.5–45 Hz) and the intergral of power of bandpass filtered recordings were calculated.

In a subgroup of animals tested with 10^−5^ mol/L SP the regional cortical perfusion (regional cortical blood flow, rCBF) was measured continuously with a flow meter (Laser Blood Flow Monitor DRT4, Moor Instruments, Millwey, Axminster, Devon, EX13 5HU, UK). A sensor with a tip diameter of approximately 1 mm was placed onto the cerebral surface in the area where SP was applied that was void of major blood vessels but near to the DC recording electrodes. Changes in regional cortical perfusion during application of SP were calculated as percentage of predrug values.

#### Application of SP and the NK-1 receptor antagonist

The compounds were administered topically onto the exposed cortex into the trough. We applied SP (Sigma-Aldrich, Germany) at either 10^−5^; 10^−6^; 10^−7^ or 10^−8^ mol/L, dissolved in aqua bidest. for two hours. In each experiment only one dose of SP was tested because binding of SP to the NK-1R causes a rapid internalisation of this receptor. Internalization may occur within minutes and resensitisation by reappearance of the NK-1R may take 2 hours^52^. The particular group sizes and protocols are given in the legends or figures. For comparison, we applied in a subset of three rats SP at 10^−5^ mol/L, dissolved in ACSF. In another group of three rats we conditioned the treated area for two hours with acetate-ACSF and then applied SP at 10^−6^ mol/L, dissolved in ACSF.

In a control group of four rats we applied only 100 µL of aqua bidest. topically and recorded the DC potentials as well. In another group of three rats we conditioned the brain with acetate-ACSF for two hours and then applied 100 µL of aqua bidest.

In order to demonstrate the specific effect of SP to the NK-1R, we applied in a group of five rats first 250 nmol/L of the NK-1RA L-703,606 (Sigma-Aldrich, Germany, dissolved in PBS) for one hour, and CSD were elicited with KCl after 30 and 60 min. Then the cortex was rinsed with ACSF and SP at 10^−5^ mol/L dissolved in aqua bidest. was applied for two hours. For comparison, we repeated this experiment in three rats after a previous conditioning of the treated brain area with acetate-ACSF. Then these rats received a pretreatment with 250 nmol/L NK-1RA for one hour followed by application of 100 µL aqua bidest. In two other rats we applied 100 µL aqua bidest. after a pretreatment with 250 nmol/L NK-1RA for one hour without acetate conditioning. At the end of the experiments the animals were sacrificed using an overdose of Trapanal (100 mg per rat, intravenously).

Data are reported as means ± Std. dev. For statistics we performed tests within the groups (Wilcoxon signed-rank test or paired Student’s t-test, when appropriate) and between groups (one-way ANOVA). When required Bonferroni adjustment was performed. Significance was accepted at p < 0.05. The exact p-values are stated in the results.

#### Assessment of plasma extravasation

In some experiments with SP at 10^−5^ mol/L, or SP at 10^−5^ mol/L after pretreatment with the NK-1RA, or with application of aqua alone, animals received 0.5 mL of 4% Evans Blue (Sigma-Aldrich, Saint Louis, USA) intravenously for the assessment of plasma extravasation after having completed the CSD recording protocol. As a control we induced a CSD by KCl 30 min after injection of the dye in order to check whether the dye could have influenced the ability to generate CSD. Evans Blue binds to albumin. Under normal conditions it will not permeate from blood vessels into the surrounding tissue. The rats were transcardially perfused with PBS buffer under deep anaesthesia until euthanasia followed by 4% ice-cold phosphate-buffered paraformaldehyde (PFA; Sigma-Aldrich). Brains were directly removed, postfixed for 24 hours in 4% PFA, equilibrated in 30% sucrose, and frozen. Then they were cut in sequential 40 µm transversal sections using the Leica CM3050S cryostat (Leica Biosystems, Nussloch, Germany), and embedded in Aqua Poly/Mount (Polysciences, Inc., Warrington, USA). Images of plasma extravasation were recorded using the confocal laser scanning microscope TCS SP5 (Leica, Wetzlar, Germany). To evoke a fluorescence signal Evans Blue was excited with a diode-pumped solid-state laser at 561 nm. Fluorescence signals were recorded between 570 and 700 nm using a 20x 0.7 dry objective.

## Data Availability

The datasets generated during and/or analysed during the current study are available from the corresponding author on reasonable request.
